# NK105, a paclitaxel-incorporating micellar nanoparticle formulation, can extend *in vivo* antitumour activity and reduce the neurotoxicity of paclitaxel

**DOI:** 10.1038/sj.bjc.6602479

**Published:** 2005-03-22

**Authors:** T Hamaguchi, Y Matsumura, M Suzuki, K Shimizu, R Goda, I Nakamura, I Nakatomi, M Yokoyama, K Kataoka, T Kakizoe

**Affiliations:** 1 Department of Medicine, President of National Cancer Center, 5-1-1 Tsukiji, Chuo-ku, Tokyo 104-0045, Japan; 2 Investigative Treatment Division, National Cancer Center Research Institute East, 6-5-1 Kashiwanoha, Kashiwa, Chiba 277-8577, Japan; 3 Pharmaceuticals Group, Research & Development Division, Nippon Kayaku Co., Ltd, 3-31-12 Shimo, Kita-ku, Tokyo 115-8588, Japan; 4 NanoCarrier Co., Ltd, Tokatsu Techno Plaza, 5-4-6 Kashiwanoha, Kashiwa, Chiba 277-0882, Japan; 5 Kanagawa Academy of Science and Technology, KSP Bldg., East 404, 3-2-1 Sakado, Takatsu-ku, Kawasaki, Kanagawa 213-0012, Japan; 6 Department of Materials Engineering, Graduate School of Engineering, The University of Tokyo, 7-3-1 Hongo, Bunkyo-ku, Tokyo 113-8656, Japan; 7 President of National Cancer Center, 5-1-1 Tsukiji, Chuo-ku, Tokyo 104-0045, Japan

**Keywords:** NK105, paclitaxel, polymer micelles, DDS, EPR effect

## Abstract

Paclitaxel (PTX) is one of the most effective anticancer agents. In clinical practice, however, high incidences of adverse reactions of the drug, for example, neurotoxicity, myelosuppression, and allergic reactions, have been reported. NK105, a micellar nanoparticle formulation, was developed to overcome these problems and to enhance the antitumour activity of PTX. Via the self-association process, PTX was incorporated into the inner core of the micelle system by physical entrapment through hydrophobic interactions between the drug and the well-designed block copolymers for PTX. NK105 was compared with free PTX with respect to their *in vitro* cytotoxicity, *in vivo* antitumour activity, pharmacokinetics, pharmacodynamics, and neurotoxicity. Consequently, the plasma area under the curve (AUC) values were approximately 90-fold higher for NK105 than for free PTX because the leakage of PTX from normal blood vessels was minimal and its capture by the reticuloendothelial system minimised. Thus, the tumour AUC value was 25-fold higher for NK105 than for free PTX. NK105 showed significantly potent antitumour activity on a human colorectal cancer cell line HT-29 xenograft as compared with PTX (*P*<0.001) because the enhanced accumulation of the drug in the tumour has occurred, probably followed by its effective and sustained release from micellar nanoparticles. Neurotoxicity was significantly weaker with NK105 than with free PTX. The neurotoxicity of PTX was attenuated by NK105, which was demonstrated by both histopathological (*P*<0.001) and physiological (*P*<0.05) methods for the first time. The present study suggests that NK105 warrants a clinical trial for patients with metastatic solid tumours.

Paclitaxel (PTX) is one of the most useful anticancer agents known for various cancers including ovarian, breast, and lung cancers (Carney, 1996; Khayat *et al*, 2000). However, PTX has serious adverse effects, for example, neutropenia and peripheral sensory neuropathy. In addition, anaphylaxis and other severe hypersensitive reactions have been reported to develop in 2–4% of patients receiving the drug even after premedication with antiallergic agents; these adverse reactions have been attributed to the mixture of Cremophor EL and ethanol, which was used to solubilise PTX (Weiss *et al*, 1990; Rowinsky and Donehower, 1995). Of the adverse reactions, neutropenia can be prevented or managed effectively by administering a granulocyte colony-stimulating factor. On the other hand, there are no effective therapies to prevent or reduce nerve damage, which is associated with peripheral neuropathy caused by PTX; therefore, neurotoxicity constitutes a significant dose-limiting toxicity of the drug (Rowinsky *et al*, 1993; Wasserheit *et al*, 1996).

The above problems of PTX have been attributed to its low therapeutic indices and limited efficacy due to the nonselective nature of its therapeutic targets and its inability to accumulate selectively in cancer tissue. Therefore, there is an urgent need to develop modalities by which cytotoxic drugs can selectively target tumour tissue and effectively act on cancer cells in the scene. The roles of drug delivery systems (DDSs) have drawn attention in this context. Drug delivery systems are based on two main principles: active and passive targetings. The former refers to the development of monoclonal antibodies directed against tumour-related molecules that allow targeting of the tumour because of specific binding between the antibody and its antigen. However, the application of DDSs using monoclonal antibodies is restricted to tumours expressing high levels of related antigens.

Passive targeting is based on the so-called enhanced permeability and retention (EPR) effect (Matsumura and Maeda, 1986; Maeda *et al*, 2000). The EPR effect consists in the pathophysiological characteristics of solid tumour tissue: hypervascularity, incomplete vascular architecture, secretion of vascular permeability factors stimulating extravasation within cancer tissue, and absence of effective lymphatic drainage from tumours that impedes the efficient clearance of macromolecules accumulated in solid tumour tissues.

Several techniques to maximally use the EPR effect have been developed, that is, modification of drug structures and development of drug carriers. The first micelle-forming polymeric drug developed was polyethylene glycol (PEG)-polyaspartate block copolymer conjugated with doxorubicin (DXR) (Yokoyama *et al*, 1990; Yokoyama *et al*, 1991; Kataoka *et al*, 1993). PEG constituted the outer shell of the micelle, which conferred a stealth property on the drug that allowed the micellar drug preparations to be less avidly taken up by the reticuloendothelial system (RES) and to be retained in the circulation for a longer time. Prolonged circulation time and the ability of polymeric micelles to extravasate through the leaky tumour vasculature were expected to result in the accumulation of DXR in tumour tissue due to the EPR effect (Kwon *et al*, 1994; Yokoyama *et al*, 1999). A clinical trail of micellar DXR, NK911, is now underway (Nakanishi *et al*, 2001; Hamaguchi *et al*, 2003). Recently, we succeeded in constructing NK105, a polymeric micelle carrier system for PTX, which conferred on PTX a passive targeting ability based on the EPR effect. In the present paper, we describe the details and characteristics of NK105. We also discuss differences between NK105 and other DDS formulations containing PTX.

## MATERIALS AND METHODS

### Materials

PTX was purchased from Mercian Corp. (Tokyo, Japan). All other chemicals were of reagent grade. Following cell lines, MKN-45, MKN-28, HT-29, DLD-1, HCT116, TE-1, TE-8, PC-14, PC-14/TXT, H460, MCAS, OVCAR-3, AsPC-1, PAN-9, PAN-3, and MCF-7 cells were purchased from American Type Culture Collection. Colon 26 cells were dispensed from the Japan Foundation for Cancer Research (Tokyo, Japan). Female BALB/c *nu/nu* mice were purchased from SLC (Shizuoka, Japan). Female CDF1 mice and IGS rats were purchased from Charles River Japan Inc. (Kanagawa, Japan).

All animal procedures were performed in compliance with the guidelines for the care and use of experimental animals, which had been drawn up by the Committee for Animal Experimentation of the National Cancer Center; these guidelines meet the ethical standards required by law and also comply with the guidelines for the use of experimental animals in Japan.

### NK105, a PTX-incorporating micellar nanoparticle formulation

NK105 is a PTX-incorporating ‘core-shell-type’ polymeric micellar nanoparticle formulation. Polymeric micellar particles were formed by facilitating the self-association of amphiphilic block copolymers in an aqueous medium. Novel amphiphilic block copolymers, namely NK105 polymers, were designed for PTX entrapment. NK105 polymers were constructed using PEG as the hydrophilic segment and modified polyaspartate as the hydrophobic segment. Carboxylic groups of polyaspartate block were modified with 4-phenyl-1-butanol by esterification reaction, consequently the half of the groups were converted to 4-phenyl-1-butanolate. Via the self-association process, PTX was incorporated into the inner core of the micelle system by physical entrapment through hydrophobic interactions between the drug and specifically well-designed block copolymers for PTX.

### Pharmacokinetics and pharmacodynamics of PTX and NK105

Colon 26 tumour-bearing CDF1 mice aged 8 weeks were given intravenously (i.v.) via the tail vein PTX 50 and 100 mg kg^−1^ or NK105 at corresponding PTX-equivalent doses. Mice were killed at 5 and 30 min, as well as 2, 6, 24, and 72 h after injection. Blood was collected, and tumours were removed; plasma and tumours obtained were then stored at −20°C until the analysis. Each time point for collection represented three samples from three different mice. PTX was extracted from plasma obtained by deproteinisation using acetonitrile, followed by liquid–liquid extraction with *t*-butylmethylether. Tumours obtained were homogenised in 0.5% acetic acid, and the resultant homogenate was deproteinised and extracted according to the same method as that used for plasma. The blood and tumour extracts were analysed for PTX by liquid chromatography/tandem mass spectrometry. Reversed-phase column-switching chromatography was conducted using an ODS column and detection was enabled by electrospray ionisation of positive mode. The mean plasma and tumour concentrations of PTX at each sampling point were calculated for both PTX and NK105. Pharmacokinetic modelling was completed using a WinNonlin Standard software version 3.1 (Pharsight Corp., California, USA).

### *In vitro* cytotoxicity

Various human cancer cell lines were evaluated in the present study. The cell lines were maintained in monolayer cultures in Dulbecco's modified Eagle's medium containing 10% (v v^−1^) foetal calf serum and 600 mg l^−1^ glutamine. WST-8 Cell Counting Kit-8 (Dojindo, Kumamoto, Japan) was used for the cell proliferation assay. In all, 2000 cells of each cell line in 90 *μ*l of culture medium were plated in 96-well plates and were then incubated for 24 h at 37°C. Serial dilutions of PTX or NK105 in a volume of 10 *μ*l were added, and the cells were incubated for 48 or 72 h. All data were expressed as mean±s.e. of triplicate cultures. The data were then plotted as a percentage of the data from the control cultures, which were treated identically to the experimental cultures, except that no drug was added.

### Evaluation of the antitumour activity of PTX and NK105

The antitumour activity of PTX and NK105 was evaluated using nude mice implanted with a human colonic cancer cell line, HT-29. One million tumour cells of HT-29 were inoculated at a subcutaneous (s.c.) site on the back skin of BALB/c female nude mice aged 6 weeks. When tumour size reached approximately 5–8 mm in diameter, mice were randomly allocated to the PTX administration group, NK105 administration group, and control administration group, each of which was made up of five animals. Each treatment was carried out as follows: free PTX group was administered at a dose of 25, 50, or 100 mg kg^−1^; NK105 group was with same PTX-equivalent doses; and in control group, animals were given saline. Mice were administered a single i.v. injection of PTX or NK105 weekly for 3 weeks. The antitumour activity of PTX and NK105 was evaluated by measuring tumour size (*a* × *b*, where *a* is the major diameter and *b* is the minor diameter) at various time points after injection. Changes in body weight were also monitored for mice, which were used in the present study.

### Evaluation of neurotoxicity

The severity of neurotoxicity was assessed both electrophysiologically and histologically. Under intraperitoneal ketamine anaesthesia (40 mg kg^−1^), rats were given a single i.v. injection of PTX (7.5 mg kg^−1^), NK105 (a PTX-equivalent dose of 7.5 mg kg^−1^), or 5% glucose weekly for 6 weeks. All the solutions were administered through the jugular vein exposed via a small incision in the neck. Electrophysiological measurements were conducted 1 day before the first dosing and on day 6 after the final dosing. For electrophysiological recording, rats were anaesthetised by the intraperitoneal injection of pentobarbital 40 mg kg^−1^. Electrical stimuli were given peripherally, and caudal sensory nerve action potentials (caudal SNAPs) were recorded centrally from the tail. The amplitude of each waveform was calculated by measuring the caudal SNAP from the top peak to the bottom peak. Variations in the amplitude after the 6th weekly administration of the solutions were determined.

For light microscopy, rats were killed after electrophysiological recordings. Subsequently, a segment of the sciatic nerve was carefully removed, and embedded in paraffin. Sections (2 *μ*m thick) were stained with haematoxylin and eosin (H & E) before examination under light microscopy to evaluate the degenerative changes of myelinated nerve fibres.

### Statistical analysis

The data of therapeutic efficacy was expressed as mean±s.e.m. The statistical significance of differences in therapeutic efficacy between two administration groups was calculated by means of repeated measures (analysis of variance). The statistical significance of the differences in neurotoxic activity between two administration groups was calculated using the Student's *t*-test on the closed testing procedure. The histopathological impairment was scored in five grades. The statistical significance of the differences in histopathological impairment between two administration groups was calculated using the Wilcoxon's rank-sum test on the closed testing procedure. All data were calculated with software StatView, version 5 (ABACUS Concepts, Berkeley, CA, USA). A value of *P*<0.05 was considered statistically significant.

## RESULTS

### Preparation and characterisation of NK105

**Figure 1:**
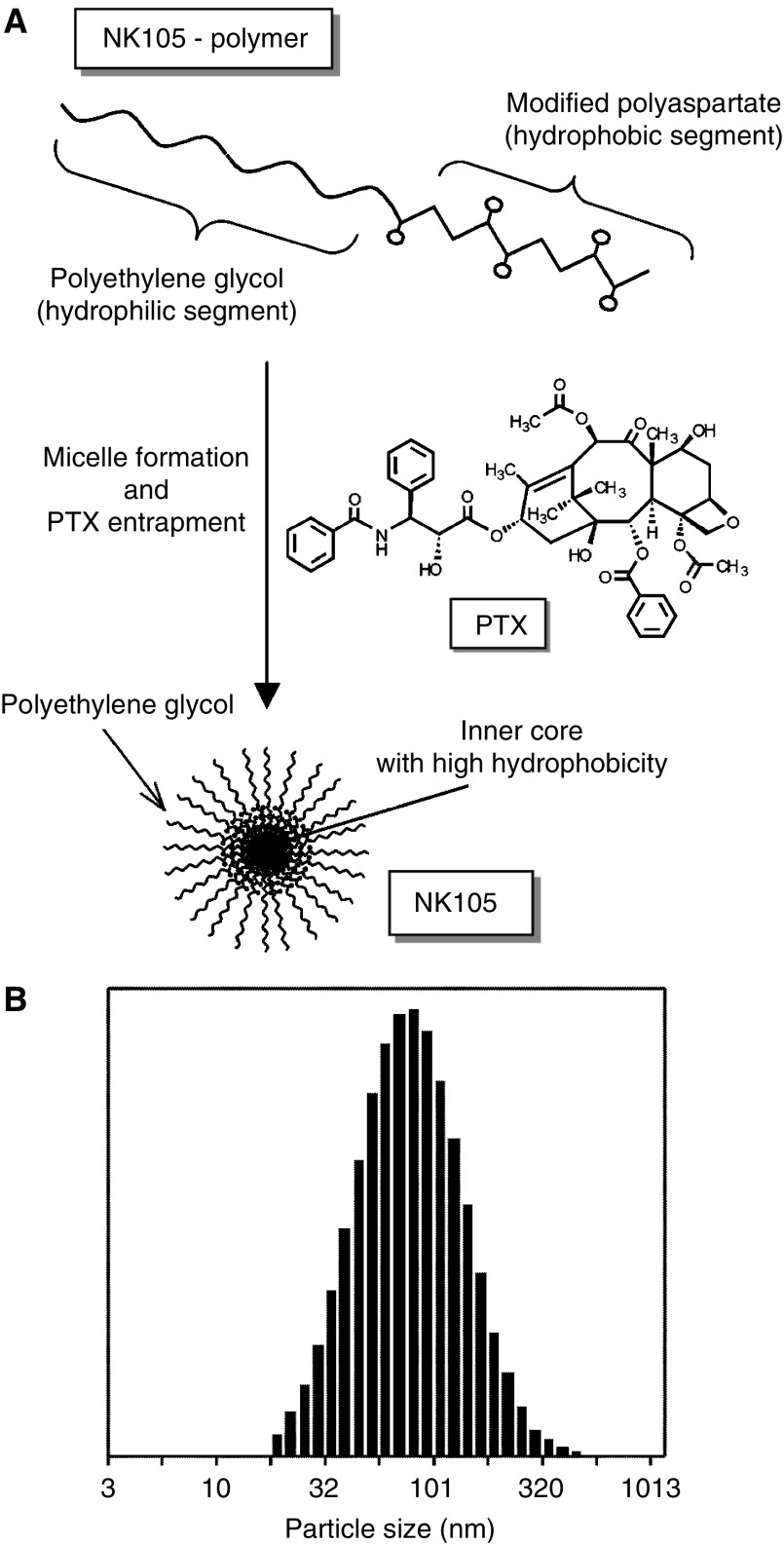
Preparation and characterisation of NK105. (**A**) The micellar structure of NK105 PTX was incorporated into the inner core of the micelle. (**B**) The size distribution of NK105 measured by the dynamic light scattering method. The mean diameter of an NK105 micelle was 85 nm.

To construct NK105 micellar nanoparticles (Figure 1A), block copolymers consisting of PEG and polyaspartate, the so-called PEG polyaspartate described previously (9, 11, 13, 14), were used. PTX was incorporated into polymeric micelles formed by physical entrapment utilising hydrophobic interactions between PTX and the block copolymer polyaspartate chain. After screening of many candidate substances, 4-phenyl-1-butanol was employed for the chemical modification of the polyaspartate block to increase its hydrophobicity. Treating with a condensing agent, 1,3-diisopropylcarbodiimide, the half of carboxyl groups on the polyaspartate, was esterified with 4-phenyl-1-butanol. Molecular weight of the polymers was determined to be approximately 20 000 (PEG block: 12 000; modified polyaspartate block: 8000). NK105 was prepared by facilitating the self-association of NK105 polymers and PTX. NK105 was obtained as a freeze-dried formulation and contained ca. 23% (w w^−1^) of PTX, as determined by reversed-phase liquid chromatography using an ODS column with mobile phase consisting of acetonitrile and water (9 : 11, v v^−1^) and detection of ultraviolet absorbance at 227 nm. Finally, NK105, a PTX-incorporating polymeric micellar nanoparticle formulation with a single and narrow size distribution, was obtained. The weight-average diameter of the nanoparticles was approximately 85 nm ranging from 20 to 430 nm (Figure 1B).

### Pharmacokinetics and pharmacodynamics of NK105

**Figure 2:**
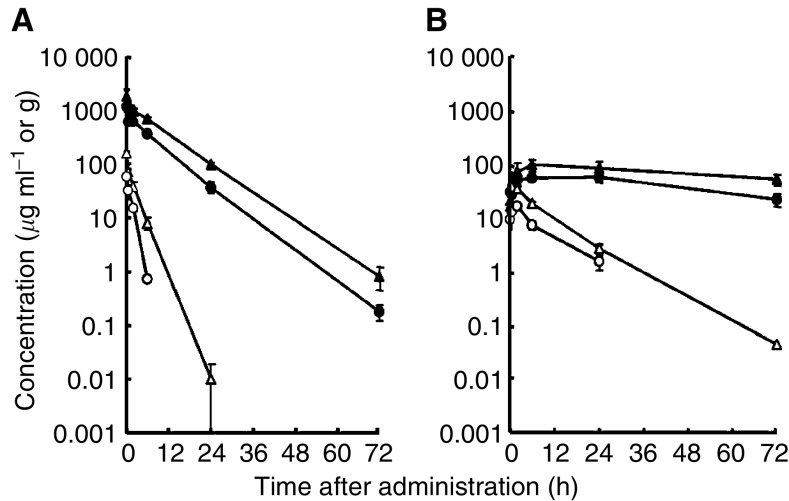
Plasma and tumour concentrations of PTX after single i.v. administration of NK105 or PTX to Colon 26-bearing CDF1 mice. Plasma (**A**) and tumour (**B**) concentrations of PTX after NK105 administration at a PTX-equivalent dose of 50 mg kg^−1^ (•), NK105 at a PTX-equivalent dose of 100 mg kg^−1^ (**▴**), PTX 50 mg kg^−1^ (○) and PTX 100 mg kg^−1^ (▵).

Colon 26-bearing CDF1 mice were given a single i.v. injection of PTX 50 or 100 mg kg^−1^, or of NK105 at an equivalent dose of PTX. Subsequently, the time-course changes in the plasma and tumour levels of PTX were determined in the PTX and NK105 administration groups (Figure 2); furthermore, the pharmacokinetic parameters of each group were also determined (Table 1). NK105 exhibited slower clearance from the plasma than PTX, while NK105 was present in the plasma for up to 72 h after injection; PTX was not detected after 24 h or later of injection. The plasma concentration at 5 min (*C*_5 min_) and the area under the curve (AUC) of NK105 were 11–20-fold and 50–86-fold higher for NK105 than for PTX, respectively. Furthermore, the half-life at the terminal phase (*t*_1/2_*z*) was 4–6 times longer for NK105 than for PTX. The maximum concentration (*C*_max_) and AUC of NK105 in Colon 26 tumours were approximately 3 and 25 times higher for NK105 than for PTX, respectively. NK105 continued to accumulate in the tumours until 72 h after injection. The tumour PTX concentration was higher than 10 *μ*g g^−1^ even at 72 h after the i.v. injection of NK105 50 and 100 mg kg^−1^. On the contrary, the tumour PTX concentrations at 72 h after the i.v. administration of free PTX 50 and 100 mg kg^−1^ were below detection limits and less than 0.1 *μ*g g^−1^, respectively.

### *In vitro* cytotoxicity

NK105 was tested on 12 human tumour cell lines derived from lung, gastric, oesophagus, colon, breast, and ovarian tumours. Similar dose–response curves were noted for PTX and NK105 (data not shown). Furthermore, the IC_50_ values of NK105 were similar to those of PTX at 48 and 72 h, indicating that both NK105 and PTX showed equivalent cytotoxic activity *in vitro* (Table 2).

### *In vivo* antitumour activity

**Figure 3:**
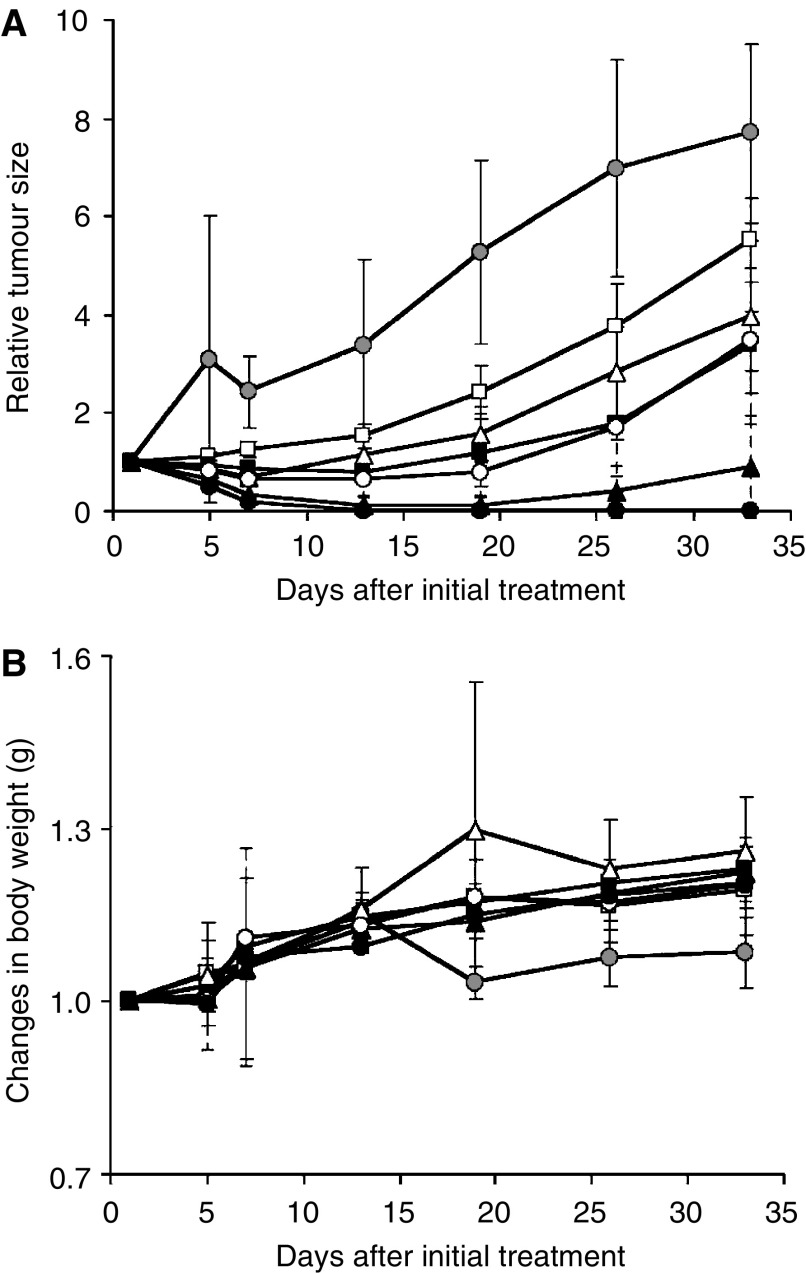
Relative changes in HT-29 tumour growth rates in nude mice. (**A**) Effects of PTX (open symbols) and NK105 (closed symbols). PTX and NK105 were injected i.v. once weekly for 3 weeks at PTX-equivalent doses of 25 mg kg^−1^ (□, ▪), 50 mg kg^−1^ (▵, ▴), and 100 mg kg^−1^ (○, •), respectively. Saline was injected to control animals (•). (**B**) Changes in relative body weight. Data were derived from the same mice as those used for the present study.

BALB/c mice bearing s.c. HT-29 colon cancer tumours showed decreased tumour growth rates after the administration of PTX and NK105. However, NK105 exhibited superior antitumour activity as compared with PTX (*P*<0.001). The antitumour activity of NK105 administered at a PTX-equivalent dose of 25 mg kg^−1^ was comparable to that obtained after the administration of free PTX 100 mg kg^−1^. Tumour suppression by NK105 increased in a dose-dependent manner. Tumours disappeared after the first dosing to mice treated with NK105 at a PTX-equivalent dose of 100 mg kg^−1^, and all mice remained tumour-free thereafter (Figure 3A). In addition, less weight loss was induced in mice, which were given NK105 100 mg kg^−1^ than in those that were given the same dose of free PTX (Figure 3B).

### Neurotoxicity of PTX and NK105

Treatment with PTX has resulted in cumulative sensory-dominant peripheral neurotoxicity in humans, characterised clinically by numbness and/or paraesthesia of the extremities. Pathologically, axonal swelling, vesicular degeneration, and demyelination were observed. We, therefore, examined the effects of free PTX and NK105 using both electrophysiological and morphological methods.

**Figure 4:**
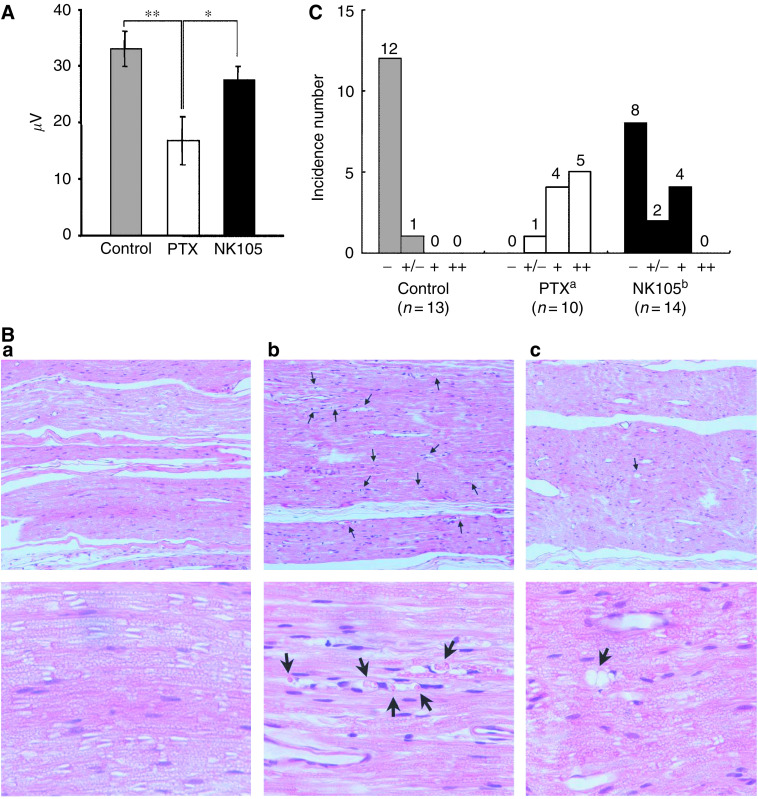
Incorporation of PTX into polymeric micelles diminishes neurotoxicity. (**A**) Effects of PTX or NK105 on the amplitude of rat caudal sensory nerve action potentials as examined 5 days after weekly injections for 6 weeks. Rats (*n*=14) were injected with NK105 (▪) or PTX (□) at a PTX-equivalent dose of 7.5 mg kg^−1^. Glucose (5%) was also injected in the same manner to animals in the control group (▪). ^*^P<0.05, **P<0.01. (**B**) Histopathological changes in the sciatic nerve of rats. Degenerating myelinated nerve fibres (arrow) were examined in the longitudinal section of the sciatic nerve (H & E) 5 days after weekly injections for 6 weeks with 5% glucose (a), PTX (b), and NK105 (c) at a PTX-equivalent dose of 7.5 mg kg^−1^. Magnification, × 100 (upper) and × 400 (lower). (**C**) Incidences of degenerating myelinated nerve fibres in rats treated with PTX or NK105. NK105 or PTX was administered i.v. at a weekly dose of 7.5 mg kg^−1^ for 6 consecutive weeks to female rats. The degenerating myelinated fibre score was defined as follows: −, no degenerative changes; +/−, very slight degree of the degenerative changes (scattered, single fibres affected); +, slight degree of degenerative changes (scattered small groups of degenerative myelinated fibres); ++, moderate degree of degenerative changes (disseminated degenerative myelinated fibres); +++, marked degree of degenerative changes (confluent groups of affected fibres). ^a^*P*<0.001 *vs* vehicle-treated animals. ^b^*P*<0.001 *vs* PTX-treated animals.

Prior to drug administration, there were no significant differences in the amplitude of caudal sensory nerve action potential (caudal SNAP) between two drug administration groups. On day 6 after the last dosing (at week 6), the amplitude of the caudal SNAP in the control group increased in association with rat maturation. The amplitude was significantly smaller in the PTX group than in the control group (*P*<0.01), while the amplitude was significantly larger in the NK105 group than in the PTX group (*P*<0.05) and was comparable between the NK105 group and the control group (Figure 4A). Histopathological examination of longitudinal paraffin-embedded sections of the sciatic nerve 5 days after the sixth weekly injection revealed degenerative changes. The NK105 administration group showed only a few degenerative myelinated fibres in contrast to the PTX administration group, which indicated markedly more numerous degenerative myelinated fibres (*P*<0.001) (Figure 4B and C) and Table 3.

## DISCUSSION

A pharmacokinetic study revealed that the plasma AUC of NK105 was approximately 90-fold higher than that of free PTX in the present rodent models. Prolonged circulation of NK105 in the blood due to the EPR effect was associated with a significant increase in the tumour AUC. In fact, the tumour AUC of NK105 was approximately 25-fold higher than that of free PTX (Figure 2B). In mice, accordingly, NK105 exhibited stronger antitumour activity than free PTX (Figure 3A). However, it is still debatable whether or not the enhanced accumulation of an anticancer drug into a tumour is sufficient in leading the drug to exert its antitumour activity *in vivo*.

Jain *et al* have reported that the convective passage of large drug molecules into the core of solid tumours could be impeded by abnormally high interstitial pressures in solid tumours. However, they also admitted that low-molecular-weight anticancer agents might be harmful to normal organs because they can leak out of normal blood vessels freely; they finally concluded that one useful strategy for evading the barriers to drug dispersion would be to inject patients with drug carriers, such as liposomes, filled with low-molecular-weight drugs (Jain, 1994). In this case, liposomes should have sufficient time to exit from the site of tumour blood vessel leakage and to accumulate at reasonably high dose levels in the surrounding interstitium. Subsequently, low-molecular-weight drugs packed within liposomes should be released gradually so that they can be dispersed throughout the tumour. However, Unezaki *et al* have used fluorescence-labelled PEG-liposomes and described that the area of highest fluorescence was located outside tumour vessels, almost all around the vessel wall, even 2 days after drug injection (Unezaki *et al*, 1996). Therefore, the study suggested that although PEG-liposomes can be delivered effectively to a solid tumour via the EPR effect, the formulation would not be distributed sufficiently to cancer cells distant from tumour vessels because liposomes are too large to scamper about in the tumour interstitium. Liposomes have been suggested to be too stable to allow the drug therein to be released easily. Therefore, PEG-liposomes have been speculated to be not so effective against cancers in which the tumour vessel network is irregular and loose because of an abundant collagen-rich matrix. Such cancers include scirrhous cancer of the stomach and pancreatic cancer. In fact, Doxil®, a PEG-liposomal DXR, is known to be effective clinically against ovarian cancer and breast cancer, both of which are characterised by a high density of tumour microvessels; however, the drug is not effective against stomach cancer and pancreatic cancer (Muggia, 2001).

There are several possible reasons why NK105 exhibited higher antitumour activity in the present study as compared with free PTX: (1) since NK105 is very stable in the circulation and exhibits a markedly higher plasma AUC than free PTX, it accumulates better in tumour tissue than does free PTX due to the EPR effect; (2) NK105 is relatively small in size (85 nm) as compared with Doxil (100 nm), thus explaining its more uniform distribution in tumour tissue and its greater accumulation in cancer cells throughout cancer tissue. Savic *et al* (2003) have recently reported that polymeric micelles could internalise into cells to localise in several cytoplasmic organelles; and (3) a polymeric micelle carrier system for a drug has the potential to allow the effective sustained release of the drug inside a tumour following the accumulation of micelles into tumour tissue. Regarding NK105 in particular, this sustained release begins at a PTX-equivalent dose of <1 *μ*g ml^−1^ (data not shown). Consequently, released PTX becomes distributed throughout tumour tissue and internalises into cancer cells to kill them.

To date, PTX preparations that are categorised to DDSs have been developed. Among them, clinical trials are currently ongoing for the following drugs: CT-2103, polyglutamate-conjugated PTX (Singer *et al*, 2003); ABI-007, PTX coated with albumin (Ibrahim *et al*, 2002); and Genexol-PM, PTX micelle in which PTX is simply solubilised (Kim *et al*, 2004). The advantage commonly shared with these dosage forms is that they are injectable i.v. without the mixture of Cremophor EL and ethanol, which potentially provoke serious allergic reactions. The block copolymer used for forming NK105 micellar nanoparticles is nonimmunogenic and is injectable i.v. without Cremorphor EL and ethanol. Therefore, this dosage form is expected to possess a clinical advantage, which is similar to that of the above PTX dosage forms. Now, what is the difference between NK105 and other PTX dosage forms? ABI-007 and Genexol-PM were found to have the AUC and tumour AUC, which are nearly comparable or rather slightly lower than those of free PTX. Furthermore, the plasma AUC and tumour AUC are 11.5- and 11.8-fold higher, respectively, for CT-2103 than for free PTX, but they are markedly low as compared with those of NK105. Respective studies have employed proper tumours and proper rodent models. However, NK105 was forecasted to have markedly high plasma and tumour AUC as compared with those of other PTX dosage forms.

Regarding the toxicity profiles, the repeated administration of NK105 to rats at 7-day intervals produced less toxic effects on peripheral nerves than free PTX. This reduced the neurotoxicity of NK105, which was demonstrated for the first time by both histopathological and physiological methods and was probably attributable to the less distribution of PTX into normal neural tissue following NK105 administration, since the volume of distribution at steady state (*V*_ss_) of NK105 was 100-fold lower than that of free PTX. Regarding bone marrow toxicity, there was no difference between PTX and NK105 when 37.5 mg kg^−1^ of PTX-equivalent dose was administered to rats weekly for 4 consecutive weeks (data not shown). These data indicate that NK105 warrants a clinical evaluation.

## Figures and Tables

**Table 1:** Pharmacokinetic parameters for the plasma and tumour concentrations of paclitaxel after single i.v. administration of NK105 and PTX to Colon 26-bearing CDF1 mice

**Treatment**	**Dose (mg kg^−1^)**	***C*_5 min_ (*μ*g ml^−1^)**	***t*_1/2_** ***z* (h)**	**AUC_0–*t*_ (*μ*g h ml^−1^)**	**AUC_0−inf._ (*μ*g h ml^−1^)**	**CL_tot_ (ml h kg^−1^)**	***V*_ss_ (ml kg^−1^)**
*Plasma*							
PTX	50	59.32	0.98	90.2^a^	91.3	547.6	684.6
PTX	100	157.67	1.84	309.0^b^	309.0	323.6	812.2
NK105	50	1157.03	5.99	7860.9^c^	7862.3	6.4	46.4
NK105	100	1812.37	6.82	15 565.7^c^	15 573.6	6.4	54.8
							

		***C*_max_ (*μ*g ml^−1^)**	***T*_max_ (h)**	***t*_1/2_** ***z* (h)**	**AUC_0–*t*_** **(*μ*g h ml^−1^)**	**AUC_0–inf._ (*μ*g h ml^−1^)**	
*Tumour*							
PTX	50	12.50	2.0	7.02	120.8^b^	133.0	
PTX	100	28.57	0.5	8.06	330.4^c^	331.0	
NK105	50	42.45	24.0	35.07	2360.1^c^	3192.0	
NK105	100	71.09	6.0	73.66	3884.9^c^	7964.5	

i.v.=intravenous; *C*_5 min_=plasma concentration at 5 min; *t*_1/2_*z*=half-life at the terminal phase; AUC=area under the curve; CL_tot_=total body clearance; *V*_ss_=volume of distribution at steady state; *T*_max_=time of maximum concentration; PTX=paclitaxel.

Parameters were calculated from the mean value of three or two mice by noncompartmental analysis.

a AUC_0-6h_.

b AUC_0–24 h_.

c AUC_0–72 h_.

**Table 2:** IC_50_ values (*μ*M) of PTX and NK105 in various cell lines

**Cancer**	**Cell line**	**48 h**		**72 h**	
		**NK105**	**PTX**	**NK105**	**PTX**
Oesophageal cancer	TE-1	>1.0	>1.0	0.01	0.02
	TE-8	0.02	0.02	0.01	0.01
					
Lung cancer	PC-14	0.01	0.01	0.01	0.01
	PC-14/TXT	0.15	0.09	0.08	0.06
	H460	ND	ND	0.03	0.01
					
Breast cancer	MCF-7	>1.0	>1.0	0.01	0.01
Stomach cancer	MKN-28	0.03	0.03	0.01	0.21
	MKN-45	0.02	0.07	0.01	0.02
					
Colon cancer	DLD-1	0.95	0.26	0.29	0.20
	HT-29	0.01	0.01	0.01	0.01
	HCT116	ND	ND	0.03	0.01
					
Ovarian cancer	MCAS	0.01	0.01	0.01	0.01
	OVCAR-3	>1.0	>1.0	>1.0	>1.0
					
Pancreatic cancer	AsPC-1	ND	ND	0.02	0.02
	PAN-9	ND	ND	0.03	0.02
	PAN-3	ND	ND	0.010	0.004

PTX=paclitaxel; ND=not done.

**Table 3:** Incidence of degenerating myelinated fibres in rats treated with PTX or NK105

**Treatment**	***n* a**	**Degenerating myelinated nerve fibre score^b^**				
		−	**+/−**	+	**++**	**+++**
Control (vehicle)	13	12	1			
PTX^c^	10		1	4	5	
NK105^d^	14	8	2	4		

PTX=paclitaxel.

Vehicle, NK105 or PTX was administered i.v. at a weekly dose of 7.5 mg kg^−1^ for 6 consecutive weeks to female rats.

a Total number of animals accounted for that experimental condition.

b Degenerating myelinated fibre score was defined as follows: −, no degenerative changes; +/−, very slight degree of the degenerative changes (scattered, single fibres affected); +, slight degree of degenerative changes (scattered small groups of degenerative myelinated fibers); ++, moderate degree of degenerative changes (disseminated degenerative myelinated fibers); +++, marked degree of degenerative changes (confluent groups of affected fibres).

c *P*<0.001 *vs* vehicle-treated animals.

d *P*<0.001 *vs* PTX-treated animals.
